# Catechol-based polydopamine modification of a dentin adhesive: effects on surface wettability, microhardness recovery, and mineral deposition

**DOI:** 10.1007/s10856-026-07132-0

**Published:** 2026-11-03

**Authors:** Lamia Singer, Marcia Borba, Kiho Cho, Negar Mahmoudi Meimand, Nickolaos Silikas, Christian Kirschneck

**Affiliations:** 1 Oral Technology, University Hospital Bonn, Bonn, Germany; 2 Polyclinic for Orthodontics, Centre for Dental, Oral and Maxillofacial Medicine, University Hospital Bonn, Bonn, Germany; 3 Division of Dentistry, School of Medical Sciences, Faculty of Biology, Medicine and Health. The University of Manchester, Manchester, UK; 4 Dental Materials Science, Division of Applied Oral Sciences and Community Dental Care, Faculty of Dentistry, The University of Hong Kong, Hong Kong SAR, PR China

## Abstract

This study aimed to evaluate the influence of polydopamine (PDA), a bioinspired catecholamine polymer, on the functional performance of a commercial etch-and-rinse dentin adhesive when incorporated at 2% and 4% (v/v). Although PDA has demonstrated promising biomimetic and mineralisation-promoting properties in various biomedical and dental applications, its effects on the physicochemical performance of commercial etch-and-rinse dentin adhesives have not been comprehensively investigated. The study, therefore, focused on the effects of PDA incorporation on degree of conversion, pH, hydrophilicity, microhardness recovery, and mineral deposition potential. PDA was synthesised via self-polymerisation in Tris buffer (pH 8.5) and incorporated into Prime & Bond Elect adhesive at 2% and 4% (v/v). The degree of conversion was analysed using Fourier-transform infrared spectroscopy before and after light-curing. Hydrophilicity was assessed via contact angle measurements of adhesive droplets on acid-etched bovine dentin surfaces at 2 and 5 s. Microhardness was evaluated on demineralized dentin at baseline, after bonding, and following 7-day ageing in artificial saliva. Scanning electron microscopy (SEM) was used to qualitatively assess surface morphology and mineral-like deposition on deeply demineralised dentin after 14 days. pH values were measured using a microelectrode pH meter. Statistical analysis was performed using ANOVA with a significance level of *p* < 0.05. The results showed that PDA incorporation did not affect polymerisation, with comparable degrees of conversion values among all groups (*p* = 0.984), and adhesive pH remained unchanged. Dentin hydrophilicity improved with PDA modification; at 5 s, contact angles decreased from 38.1 ± 4.7° (control) to 29.8 ± 1.7° (2% PDA) and 22.5 ± 3.6° (4% PDA), demonstrating a concentration-dependent increase in wettability. Microhardness decreased after etching in all groups (*p* < 0.05), followed by partial recovery in the PDA-modified groups after ageing in artificial saliva. SEM revealed minimal surface deposition in the control, moderate mineral-like deposits in the 2% PDA group, and more extensive mineral-like surface deposition in the 4% PDA group. EDX confirmed the presence of calcium and phosphorus within the deposits, supporting their calcium phosphate-containing nature. It was found that incorporation of PDA, particularly at 4%, enhanced dentin surface hydrophilicity, promoted calcium phosphate-containing surface deposition, and improved microhardness recovery. Polymerisation efficiency and adhesive acidity were not adversely affected. These findings suggest that PDA is a promising biomimetic modifier for commercial dentin adhesives. However, further bond strength, long-term ageing, and clinical studies are needed to confirm its clinical potential.

**Figure:**
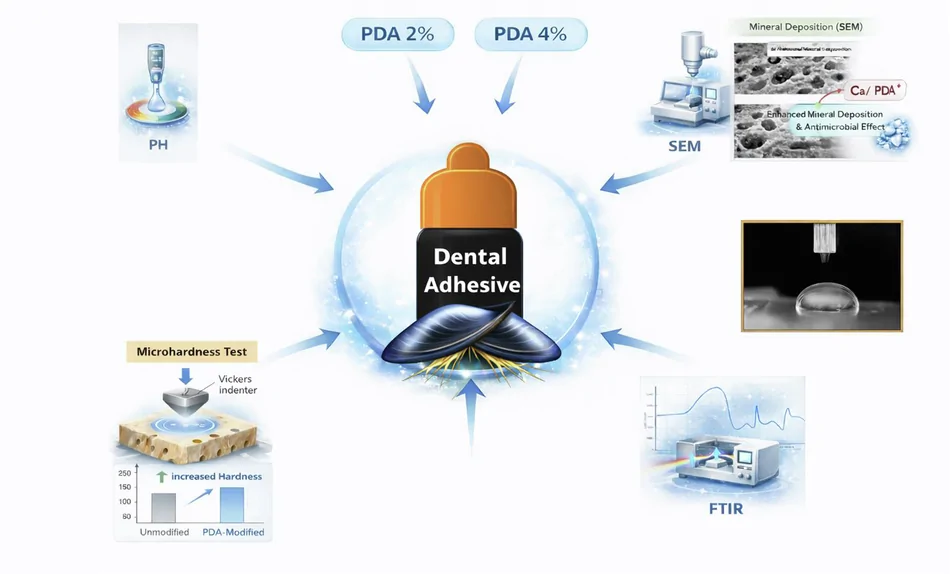

## Introduction

Bonding plays a crucial role in adhesive dentistry, occurring at the resin-dentin interface through the demineralisation of dentin [1]. This process exposes collagen fibrils, facilitating the infiltration of resin into the demineralised collagen network, ultimately forming a durable resin-dentin hybrid layer. However, the biodegradation of these resin-dentin bonds increases the risk of bacterial microleakage, which can compromise bond integrity and lead to restoration failure [2]. Compared with resin–enamel bonds, resin–dentin bonds exhibit reduced durability due to the complex structure and higher organic content of dentin [3]. Degradation of the hybrid layer may result in microleakage, staining, recurrent caries, and postoperative sensitivity, ultimately contributing to restoration failure [4–6]. Consequently, considerable research has focused on strategies to improve bond stability and longevity, including approaches aimed at enhancing interfacial resistance to degradation and reducing bacterial infiltration [7, 8].

Several studies have attempted to enhance adhesive systems by incorporating bifunctional agents with antimicrobial, anti-proteolytic, and remineralising properties [9, 10]. These modifications aimed to transform the adhesive from a passive mechanical connector into an active therapeutic interface capable of self-repair and biological defence. Reported approaches include the integration of anti-matrix metalloproteinase agents [11], calcium phosphate-based fillers [12], bioactive glass [13], and natural polymers with antibacterial functionality [14]. While these multifunctional systems are designed to disrupt the degradation cycle and prolong the service life of resin-based restorations, existing strategies still face limitations that warrant further innovation. On the other hand, silver-based agents and chitosan have also shown antibacterial potential in dental adhesives, but concerns regarding colour stability, biocompatibility, and bond strength at higher concentrations persist [15–18].

Furthermore, although protease inhibitors and collagen cross-linkers can reduce enzymatic degradation of the collagen matrix, they are unable to restore mineral content within the demineralised hybrid layer [19]. Concurrently, acids produced by cariogenic bacteria continue to promote demineralisation of both the tooth structure and restoration margins [19, 20]. These challenges highlight the need for new biomimetic formulations capable of achieving durable antimicrobial protection, mineral deposition, and biocompatibility. Therefore, an emerging and promising strategy for enhancing the long-term stability of resin–dentin bonds involves the mineral deposition of the hybrid layer [19]. Adhesive systems with mineral deposition capabilities are believed to enhance the longevity of restorations by facilitating the healing of microcracks and neutralising the acidic microenvironment within the adhesive–dentin interface [6, 21].

Demineralisation is primarily driven by a sustained low pH, which disrupts the mineral balance and weakens the collagen matrix [22]. To counteract this process, the incorporation of materials that release or promote the growth of alkaline ions, particularly calcium (Ca²⁺) and phosphate (PO₄³⁻), can help neutralise acidity and promote mineral deposition [23]. Commercially available fluoride-releasing adhesives have demonstrated reparative potential by supporting the growth of fluorapatite crystals within interfacial gaps, thereby reducing bond degradation and microleakage. In addition to fluoride, bioactive fillers such as bioactive glass, calcium phosphate compounds, and hydroxyapatite have been explored as effective sources of demineralising ions. These materials not only contribute to the mechanical reinforcement of the hybrid layer but also improve the biological performance of the adhesive by re-establishing mineral content and enhancing the stability of the resin–dentin interface [6, 24].

Polydopamine (PDA), a mussel-inspired catecholamine polymer, has recently emerged as a promising additive in adhesive dentistry due to its unique combination of adhesive, antibacterial, and mineral deposition properties [25]. Derived from the oxidative self-polymerisation of dopamine, PDA contains abundant catechol and amine functional groups that facilitate strong adhesion to both organic and inorganic surfaces, including demineralised dentin [26]. Its high surface reactivity and hydrophilicity enhance the wetting of dentin by adhesives, improving resin infiltration into the exposed collagen matrix [27]. In addition to its adhesive benefits, PDA possesses intrinsic antimicrobial properties by disrupting microbial adhesion and biofilm formation, creating a less hospitable environment for cariogenic bacteria [28]. Furthermore, PDA’s catechol groups have a high affinity for calcium and phosphate ions, allowing them to act as a nucleation platform for hydroxyapatite growth [29].

Despite its promising bioactivity, the incorporation of polydopamine directly into commercial dentin adhesives and its impact on their physicochemical behaviour remains insufficiently characterised. Therefore, this study aimed to evaluate the effect of incorporating polydopamine (PDA) into a commercial dentin bonding agent at concentrations of 2% and 4% (v/v) on its physicochemical properties and functional performance. The 2% and 4% v/v concentrations were selected based on preliminary optimisation experiments in which a range of concentrations was evaluated. Lower concentrations demonstrated minimal influence on the antimicrobial properties, whereas higher concentrations showed a tendency to adversely affect adhesive performance, particularly in terms of bond strength. Therefore, 2% and 4% were chosen as intermediate concentrations to balance potential bioactive effects with preservation of adhesive functionality.

The evaluated parameters included degree of conversion, surface hydrophilicity, mineral deposition potential, microhardness, and pH stability, along with morphological assessment to identify surface and structural changes associated with PDA modification. The null hypothesis tested was that the incorporation of PDA at 2% and 4% (v/v) would not result in significant differences in these properties or in interfacial morphology compared with the unmodified adhesive.

## Materials and methods

### Materials

Dopamine hydrochloride, Tris(hydroxymethyl)aminomethane (Tris) buffer, potassium chloride, calcium chloride, sodium chloride, sodium phosphate, and sodium azide were all purchased from Sigma-Aldrich (Steinheim, Germany). The commercial dentin bonding agent Prime & Bond Elect was obtained from Dentsply Sirona (Bensheim, Germany).

### Methods

#### Sample size considerations

An a priori power analysis was performed using G*Power software based on pilot contact angle measurements comparing the 2% and 4% PDA groups. The pilot data indicated a large effect size (Cohen’s *d* = 2.05). Based on this effect size and using a two-tailed independent-samples *t*-test (α = 0.05, power = 80%), the minimum required sample size was estimated as five specimens per group. To improve the robustness of the analysis and account for potential specimen loss, 10 specimens per group were included. Contact angle at 5 s was selected as the primary outcome. Degree of conversion (*n* = 8), microhardness (*n* = 10), and pH (*n* = 7) were evaluated as secondary outcomes. The sample sizes for these assays were selected according to the methodological requirements of each experimental technique and are consistent with previous in vitro studies. SEM/EDX analysis was performed qualitatively.

#### Preparation of polydopamine-modified dentin bonding agent

Polydopamine (PDA) was synthesised by dissolving DA hydrochloride (2 mg/mL) in Tris(hydroxymethyl)aminomethane (Tris) buffer (10 mM, pH 8.5). The solution was stirred continuously at room temperature for 24 h in darkness to allow self-polymerisation of PDA, leading to the formation of a stable PDA dispersion. The resulting PDA dispersion was filtered through a 0.45-µm syringe filter to remove any large aggregates and undissolved particles before storage at 4 °C.

The commercial dentin bonding agent Prime & Bond was modified by incorporating 2% and 4% (v/v) polydopamine (PDA). These concentrations were selected following preliminary optimisation experiments evaluating PDA concentrations ranging from 0.5% to 5% (v/v). The 2% and 4% formulations demonstrated the most favourable balance between antimicrobial performance and shear bond strength while maintaining acceptable handling characteristics and formulation homogeneity, and were therefore selected for the present study. The PDA dispersion was added to the adhesive and homogenised using a vortex mixer immediately before use to promote uniform dispersion and minimise particle aggregation. All preparation procedures were conducted under reduced light conditions to minimise premature polymerisation. The modified adhesive formulations were stored in dark, airtight containers and vortex-mixed immediately before each use to maintain homogeneity.

Three experimental groups were included in the study:

- Control Group: Unmodified adhesive.
- 2% PDA Group: Adhesive agent modified with 2% PDA.
- 4% PDA Group: Adhesive agent modified with 4% PDA.

#### Degree of conversion (DC) analysis

The polymerisation efficiency of the adhesive formulations was assessed using Fourier Transform Infrared Spectroscopy (FTIR, Bruker Tensor 27, Germany). A small drop of each adhesive formulation was placed directly on the diamond crystal of the FTIR-ATR module, forming a thin film approximately 0.1–0.2 mm thick. Spectra were acquired both before and after light curing to determine the degree of conversion (DC). Curing was performed using an LED light-curing unit (3 M ESPE Eliper DeepCure-L, Germany) with an intensity of 1200 mW/cm² for 20 s. Post-curing spectra were collected immediately after completion of the light-curing cycle using the same specimen position and acquisition parameters. The degree of conversion was calculated from the change in the aliphatic C=C absorption band at 1636 cm^−1^, normalized to C=O at 1720 cm^−1^. The DC was calculated according to the formula [30]:

1 $${{\rm{DC}}}=\left[1-\frac{{\left({A}_{\complement =\complement }/{A}_{\complement =O}\right)}_{t}}{{\left({A}_{\complement =\complement }/{A}_{\complement =O}\right)}_{0}}\right]\times 100$$

where AC=CA_{C=C}AC=C and AC=OA_{C=O}AC=O represent the absorbance intensities of the aliphatic C=C band at 1636 cm^−1^ and the carbonyl C=O band at 1720 cm^−1^, respectively. The subscript 000 denotes the uncured state, whereas t denotes the post-irradiation state measured after 20 s of light curing. Eight independent specimens were analysed per group to reduce variability associated with spectroscopic measurement [30].

#### Hydrophilicity assessment

The hydrophilicity of the bonding agent modified with 2% and 4% polydopamine was assessed by contact angle measurements on dentin surfaces. Extracted bovine anterior teeth were obtained from a commercial supplier. Following extraction, the teeth were stored in distilled water at 4 °C and used within 6 months to minimise structural degradation. Prior to use, all specimens were visually inspected, and teeth exhibiting cracks, structural defects, surface irregularities, or signs of degradation were excluded. To ensure standardisation, dentin surfaces were prepared using 600-grit silicon carbide paper under running water to obtain flat and uniform surfaces. Bovine dentin was selected due to its availability and suitability for standardised specimen preparation in in vitro studies.

The extracted bovine teeth were embedded in acrylic resin blocks, and the superficial enamel was removed using a low-speed handpiece, followed by polishing with 600-grit silicon carbide paper to expose flat dentin surfaces (5 × 5 mm). The dentin was then etched with 37% phosphoric acid for 15 s, rinsed thoroughly with deionised water, and air-dried [9]. For each specimen, a 10 µL droplet of the corresponding bonding agent formulation (control: unmodified Prime & Bond; 2% PDA-modified; or 4% PDA-modified) was dispensed vertically onto the etched dentin surface using a calibrated micropipette positioned perpendicular to the surface to ensure standardised application.

Video recording was initiated during the droplet placement and continued for 10 s after contact, using a Canon T3i (Canon Corp., USA) digital single-lens reflex camera equipped with a Canon EF 100 mm macro lens, mounted on a tripod, with horizontal alignment verified using a lever rule. Still images were extracted from the recorded videos at 2 and 5 s after droplet contact (*n* = 10). The 2-s time point was selected to evaluate the initial wetting behaviour, whereas the 5-s time point was chosen to assess early dynamic spreading following initial stabilisation of the droplet. These early measurements provide information on the interaction between the adhesive and the acid-etched dentin surface during the clinically relevant application period, when adhesive wetting and infiltration occur rapidly. A recording period of 10 s was considered sufficient to capture the initial spreading phase under the standardised experimental conditions.

**Fig. 1:**
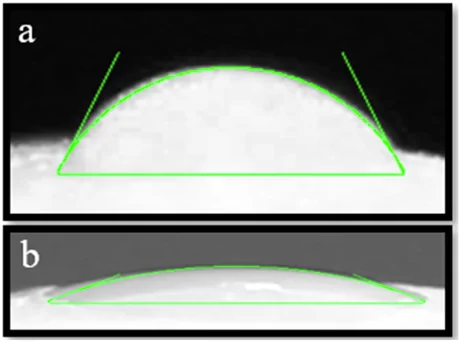
Measuring the contact angle with Image J for two different groups, **a** Control group, **b** 4%PDA group

Although a dedicated contact-angle goniometer provides higher temporal resolution, the same standardised imaging protocol and analysis procedure were applied to all groups, allowing reliable comparative evaluation of wettability. The contact angle, defined as the angle between the tangent at the droplet boundary and the dentin surface, was measured using Image J software (National Institutes of Health, USA) (Fig. 1). Lower contact angle values indicated higher hydrophilicity of the adhesive. Measurements were performed on ten specimens per group to evaluate the influence of polydopamine incorporation on the surface wettability of the bonding agent [31].

#### Assessment of mineral deposition potential

##### A. Microhardness evaluation

Extracted bovine teeth were sectioned using a low-speed diamond saw to obtain standardised dentin slabs measuring approximately 2 × 2 × 1 mm (*n* = 10). The dentin surfaces were polished with 600-grit silicon carbide paper under running water to produce flat, uniform surfaces suitable for testing. The baseline microhardness of sound dentin was measured using a Vickers microhardness tester (HMV-G, ZwickRoell, Ulm, Germany) by applying a 50 g load for 15 s at three randomly selected, non-overlapping locations on each specimen. The mean Vickers Hardness Number (VHN) was calculated for each sample. To simulate the clinical etch-and-rinse bonding protocol, dentin specimens were etched with 37% phosphoric acid for 15 s, rinsed thoroughly with deionised water, and gently air-dried to obtain a moist dentin surface before adhesive application, following the manufacturer’s instructions and established adhesive protocols [9]. Following etching, each specimen was treated with one of the adhesive formulations (control, 2% PDA-modified, or 4% PDA-modified Prime & Bond), gently air-thinned, and light-cured for 20 s using an LED curing unit with an intensity of 1200 mW/cm² [32, 33].

After adhesive application, microhardness was measured again to assess the immediate mechanical reinforcement provided by the bonding agents. Subsequently, specimens were individually immersed in 5 mL of artificial saliva (pH 7.4) and stored at 37 °C for 7 days to allow mineral deposition. The artificial saliva was composed of potassium chloride (1.5 mM), calcium chloride (1.1 mM), sodium chloride (130 mM), sodium phosphate (20 mM), and sodium azide (0.2 mM), and the solution was refreshed daily. The pH and temperature were monitored regularly throughout the storage period to maintain consistent conditions. After 7 days, microhardness testing was repeated for the final evaluation. The mean VHN values at each stage (baseline, post-etching and bonding, and post-ageing) were recorded and compared among groups. An increase in VHN after artificial saliva immersion was considered indicative of mechanical recovery and potential mineral deposition; however, this does not confirm true mineral deposition [32, 33]. Because dentin hardness varies between specimens, each sample was measured longitudinally, allowing each specimen to serve as its own baseline control.

##### B. Surface morphology and elemental analysis (SEM/EDX)

To assess mineral deposition on a more severely demineralised substrate, dentin discs (approximately 4 mm in diameter and 1 mm thick) were prepared from extracted bovine teeth using a low-speed diamond saw and polished with 600-grit silicon carbide paper. A more aggressive demineralisation protocol was applied using 37% phosphoric acid for 2 min to create a severely demineralised dentin substrate with extensive mineral depletion and exposed collagen fibrils. Unlike the clinically relevant 15-s etching protocol used for the microhardness and wettability experiments, the prolonged etching time was intentionally selected to maximise mineral removal and generate a challenging substrate for evaluating the mineral deposition potential of the experimental adhesives. This approach was designed to facilitate the detection of newly formed surface mineral deposits following immersion in artificial saliva rather than to simulate clinical adhesive procedures or the characteristics of caries-affected dentin.

After rinsing and air-drying, the discs were treated with one of the adhesive formulations (control, 2% PDA-modified, or 4% PDA-modified bonding agent) and stored in artificial saliva (pH 7.4) for 14 days at 37 °C. The saliva was replaced daily to maintain ionic strength and pH stability. Following incubation, specimens were rinsed with deionised water, air-dried, and prepared for analysis. Samples were sputter-coated with a thin layer of platinum under vacuum and examined using scanning electron microscopy (SEM) (Philips XL 30, Philips, Eindhoven, the Netherlands). Two specimens per group were examined, and representative SEM images were obtained from multiple regions of each specimen under standardized imaging conditions for qualitative assessment of surface morphology and mineral-like surface deposits [34].

To complement the morphological observations, energy-dispersive X-ray spectroscopy (EDX) was performed using an EDAX Genesis system (EDAX Inc., Mahwah, NJ, USA) coupled to the SEM. Representative surface deposits from each experimental group were analysed to determine their elemental composition. The atomic percentages of carbon (C), oxygen (O), calcium (Ca), and phosphorus (P) were recorded, and the atomic Ca/P ratio was calculated to provide compositional information regarding the calcium phosphate-containing deposits. EDX analysis was performed as a qualitative compositional assessment to confirm the presence of calcium and phosphorus within the surface deposits observed by SEM.

#### pH measurement of bonding agent formulations

The pH of the adhesive formulations was measured using a digital pH meter equipped with a small electrode designed for small-volume and viscous samples (Mettler Toledo InLab Micro). For each of the three tested groups, 100 µL of the adhesive was placed into sterile Eppendorf microcentrifuge tubes (1.5 mL capacity). The pH measurement was performed by carefully inserting the microelectrode directly into the adhesive sample inside the tube, ensuring minimal disturbance and avoiding contact with the tube walls. The electrode was allowed to stabilise before recording each measurement and was thoroughly rinsed with deionised water and gently blotted dry between measurements to prevent sample carry-over. The pH meter was calibrated immediately before measurements using standard buffer solutions at pH 4.0, 7.0, and 10.0. All measurements were carried out at room temperature and repeated seven times independently for each group. The mean pH values were recorded and used for comparative analysis of the adhesive formulations [35].

#### Statistical analysis

All experimental data were analysed using SPSS software (version 26.0, IBM, USA). Data normality was assessed using the Shapiro–Wilk test. For normally distributed data, one-way ANOVA followed by Tukey’s post hoc test was used to compare differences among groups for degree of conversion, contact angle, and pH measurements. Microhardness data were analysed using repeated-measures ANOVA to account for within-specimen comparisons across time points (baseline, post-etching, and post-ageing). SEM analysis was qualitative and not included in statistical comparisons. A significance level of *p* < 0.05 was considered statistically significant.

## Results

### Degree of conversion (DC) analysis

**Fig. 2:**
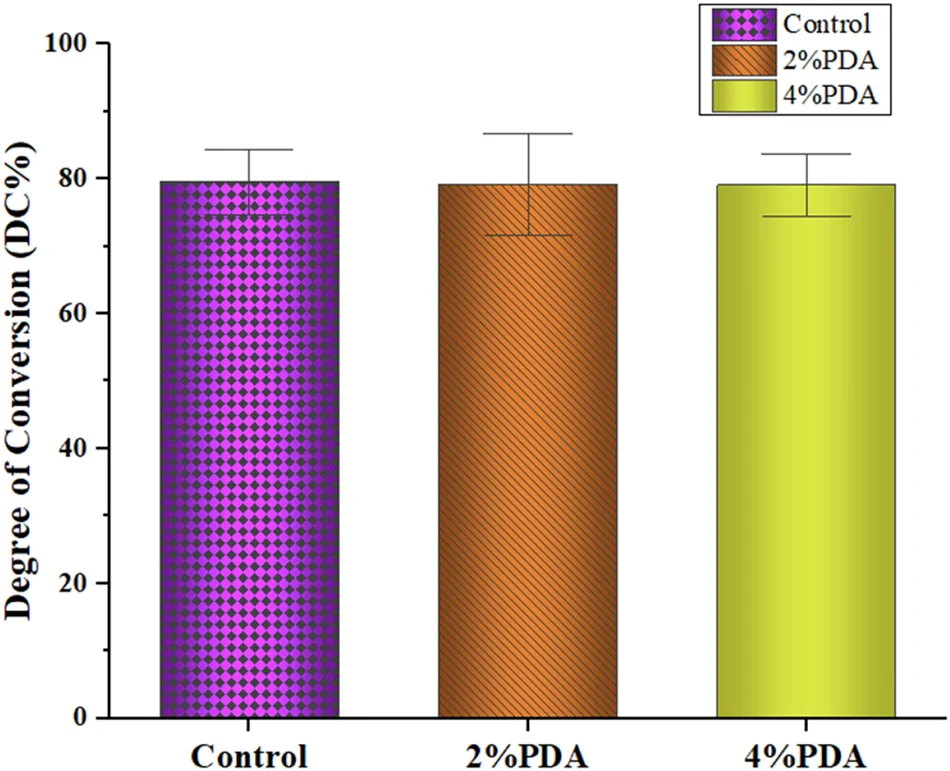
Mean ± SD of degree of conversion (%) for all tested groups. No significant differences were observed among groups

Descriptive statistics showed comparable mean DC% values among the three adhesive groups: control (79.6 ± 4.8), 2% PDA (79.2 ± 7.6), and 4% PDA (79.0 ± 4.7). Normality testing using the Shapiro–Wilk test confirmed that all groups followed a normal distribution (*p* > 0.05). Consequently, a one-way ANOVA was performed to assess statistical differences among the groups, revealing no significant effect of PDA modification on DC% (*F* = 0.0159, *p* = 0.984). These findings indicate that the incorporation of 2% or 4% polydopamine into the adhesive system did not significantly alter the degree of conversion under the tested conditions (Fig. 2). Moreover, all groups demonstrated comparable degrees of conversion under the tested conditions.

### Hydrophilicity (contact angle) measurement

**Fig. 3:**
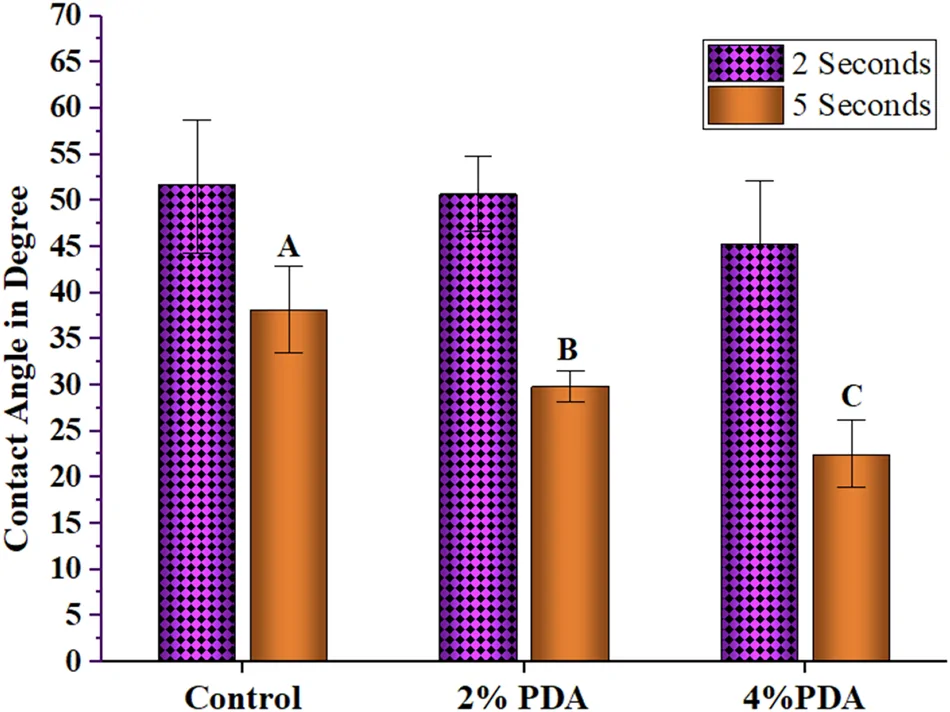
Mean ± SD of contact angle (°) after 2 and 5 s for all groups. No significant differences were found at 2 s. At 5 s, higher PDA concentrations reduced the contact angle, indicating greater hydrophilicity. Different letters (A–C) denote significant differences among groups

Contact angle analysis revealed a significant improvement in dentin surface hydrophilicity following polydopamine (PDA) modification (Fig. 3). At 2 s, the control group displayed a contact angle of 51.6 ± 7.2°, the 2% PDA group measured 50.7 ± 4.2°, and the 4% PDA group measured 45.2 ± 6.9°, with no statistically significant differences among groups. Although a decreasing trend in contact angle was observed with increasing PDA concentration, the differences did not reach statistical significance (*p* = **0.063**). At 5 s, a significant reduction in contact angle was recorded after PDA modification. The control group showed the highest mean value (38.1° A ± 4.7), while the 2% PDA group demonstrated lower contact angle values (29.8° B ± 1.7), and the 4% PDA group exhibited the lowest (22.5° C ± 3.6). The differences between all groups were statistically significant (*p* < 0.001), with a clear dose-dependent improvement in surface hydrophilicity.

### Microhardness evaluation (mechanical recovery)

**Fig. 4:**
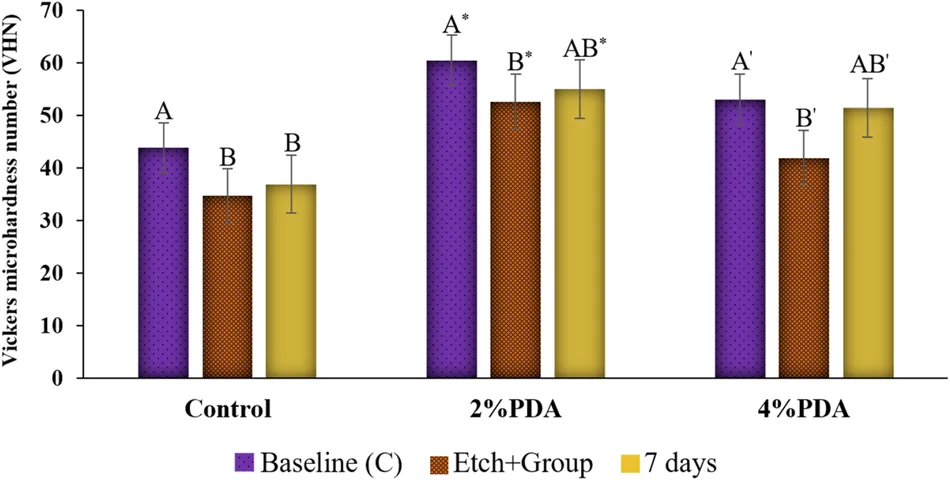
Mean ± SD of Vickers microhardness number (VHN) of dentin at baseline (C), after acid etching (Etch + Group), and following 7 days of storage in artificial saliva for the control, 2% PDA, and 4% PDA groups. Different letters indicate statistically significant differences for different conditions only within the same group

The Vickers microhardness (VHN) of dentin was measured on the same specimens at three time points: baseline, after acid etching and conditioning, and following 7 days of storage in artificial saliva. Data were analysed using repeated-measures ANOVA with Tukey’s post hoc test (*p* < 0.05). All groups showed a significant decrease in VHN after etching compared with baseline (*p* < 0.05), confirming the demineralising effect of phosphoric acid. After 7 days, partial recovery of hardness was observed in the PDA-modified groups but not in the control (Fig. 4).

In the control group, microhardness decreased significantly from 45.66 ± 5.64 VHN to 34.69 ± 7.00 VHN (*p* = 0.0005) and remained significantly lower than baseline after 7 days (*p* = 0.019). In the 2% PDA group, values decreased from 60.3 ± 9.5 VHN to 50.8 ± 6.02 VHN (*p* = 0.02) and increased to 53.51 ± 6.94 VHN after 7 days, showing no significant difference from either baseline or etched conditions (*p* > 0.05). The 4% PDA group followed a similar pattern, with hardness decreasing from 55.8 ± 8.7 VHN to 45.5 ± 9.2 VHN (*p* = 0.0089) and increasing to 51.9 ± 6.6 VHN after 7 days, again without significant difference from baseline or etched values (*p* = 0.892 and *p* = 0.246, respectively).

Both PDA-modified groups (2% and 4%) exhibited the same statistical pattern, a significant reduction after etching followed by a non-significant difference from baseline after 7 days, indicating partial recovery of dentin hardness. Numerically, an increase in the microhardness after ageing was observed in the 4% PDA group than in the 2% PDA group, although this difference was not statistically significant.

### Mineral deposition on deeply demineralised dentin

**Fig. 5:**
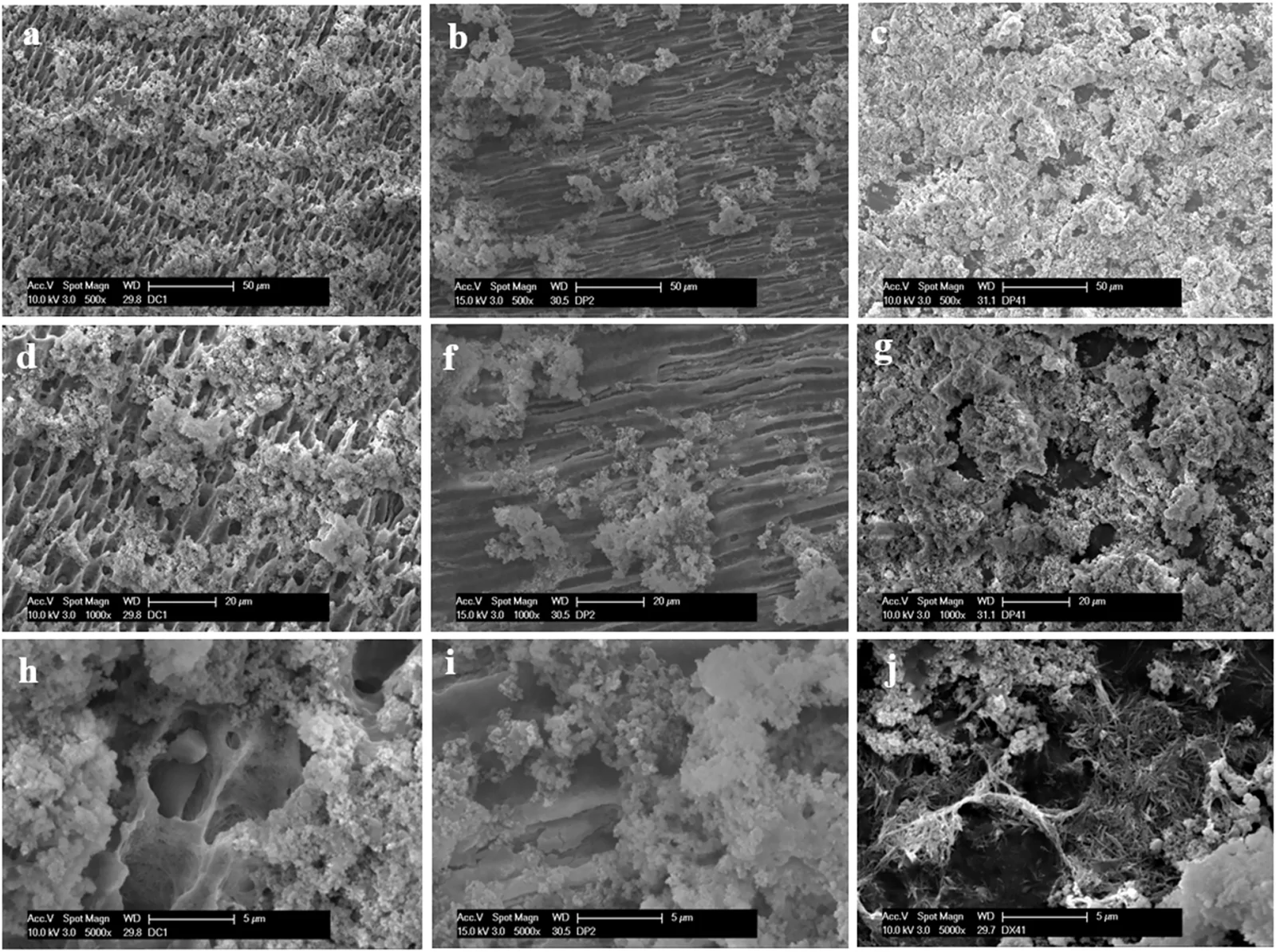
SEM images of the dentin surface after deep demineralisation, adhesive application (control, 2% PDA, and 4% PDA), and 14-day immersion in artificial saliva (pH 7.4). Images were taken at increasing magnifications: **500×** (top row), **1000×** (middle row), and **5000×** (bottom row). **a, d, h** Control group showing open dentinal tubules with minimal surface deposition. **b, f, i** 2% PDA group showing moderate surface coverage and granular mineral-like deposits along tubule walls. **c, g, j** 4% PDA group demonstrating extensive deposition with dense, irregular mineral-like structures partially occluding the tubules and covering the intertubular dentin

SEM analysis of the dentin surface after treatment with the control, 2% PDA-modified, and 4% PDA-modified adhesives revealed distinct differences in mineral deposition and surface morphology following 14 days of ageing in artificial saliva (Fig. 5). In the control group, the demineralised dentin surface remained largely porous and irregular, with open tubules and minimal evidence of mineral precipitation, indicating limited mineral deposition under the tested conditions. The 2% PDA group exhibited partial occlusion of dentinal tubules and scattered granular deposits along the surface, suggesting moderate mineral interaction. In contrast, the 4% PDA group demonstrated substantial mineral accumulation, with extensive granular and fibrillar structures resembling crystal-like mineral deposits bridging across the tubules and covering the intertubular dentin.

These deposits appeared denser and more continuous compared to the lower-concentration and control groups. The enhanced mineral deposition observed in the PDA-modified groups, particularly at 4%, is consistent with a potential role of polydopamine in facilitating mineral nucleation and growth under demineralising conditions. However, these findings reflect surface mineralisation patterns and indicate mineral deposition potential rather than definitive intrafibrillar mineral deposition.

#### EDX analysis

Representative EDX analysis confirmed the presence of calcium and phosphorus within the surface deposits in all groups (Table 1). Compared with the control, both PDA-modified groups exhibited higher calcium and phosphorus contents. The 4% PDA specimen demonstrated the highest calcium (9.55 at.%) and phosphorus (8.62 at.%) contents, followed by the 2% PDA specimen (7.11 and 5.01 at.%, respectively), whereas the control exhibited the lowest values (5.20 and 4.56 at.%). These findings support the increased formation of calcium phosphate-containing surface deposits in the PDA-modified groups, consistent with the SEM observations.

**Table 1:** Elemental composition (atomic %) and Ca/P ratio of surface deposits determined by EDX analysis of representative specimens

Group	Carbon (at.%)	Oxygen (at.%)	Calcium (at.%)	Phosphorus (at.%)	Ca/P
Control	57.88	32.36	5.20	4.56	1.14
2% PDA	48.10	38.66	7.11	5.01	1.42
4% PDA	41.91	39.92	9.55	8.62	1.11

Values represent descriptive EDX measurements obtained from representative specimens. No statistical analysis was performed.

### pH of adhesive formulations

The pH values of the adhesive formulations reflected the inherent acidity of the base adhesive. The unmodified Prime & Bond adhesive exhibited an acidic pH of 1.9 ± 0.05, consistent with its etch-and-rinse function. The incorporation of the bioinspired catechol polymer (PDA) slightly influenced the pH. The 2% PDA-modified adhesive showed a modest increase in pH to 2.0 ± 0.04, while the 4% PDA-modified adhesive exhibited a pH of 2.1 ± 0.03, remaining close to the control. These small numerical differences fall within the typical measurement variability of the pH instrumentation and therefore represent minimal practical change in acidity. Overall, PDA incorporation did not meaningfully alter the acidic nature of the bonding system.

## Discussion

This study investigated the influence of polydopamine (PDA) modification on the physicochemical and mineral deposition-related properties of an etch-and-rinse dentin adhesive. The selection of 2% and 4% PDA concentrations was based on achieving a balance between measurable interfacial modification and preservation of adhesive integrity. Preliminary optimisation experiments, supported by parallel investigations currently under review, indicated that lower concentrations produced minimal effects, whereas higher concentrations were associated with a tendency toward reduced adhesive performance, particularly in terms of bond strength. This may be attributed to increased viscosity, potential phase instability, or interference with polymerisation and resin infiltration at higher additive levels. These observations emphasise the importance of concentration optimisation when incorporating functional additives into adhesive systems.

The results consistently demonstrated that PDA incorporation, particularly at 4%, improved hydrophilicity, enhanced mineral deposition behaviour, and promoted mineral deposition on etched dentin surfaces, while maintaining clinically acceptable polymerisation behaviour and adhesive acidity. Consequently, the null hypothesis was rejected, as PDA modification produced significant changes in the evaluated properties compared with the unmodified adhesive.

The degree of conversion (DC%) is a key determinant of the polymerisation efficiency and contributes to the mechanical and physical performance of light-cured dental adhesives, as it reflects the proportion of monomeric double bonds converted into stable single bonds during polymerisation [30, 36]. However, a clinically acceptable degree of conversion does not necessarily guarantee optimal mechanical performance, as properties such as microhardness are also influenced by the polymer network architecture, crosslink density, and material composition [36]. In the present study, no statistically significant differences were observed among the tested groups (*p* ≈ 0.984), and all values remained within the clinically acceptable range for polymerisation efficiency. Although methacrylate-based adhesives may undergo limited post-irradiation polymerisation (dark curing), all formulations were analysed immediately after the 20-s light-curing cycle using an identical protocol. Therefore, any contribution of dark curing would be expected to affect all groups similarly and is unlikely to have influenced the comparative analysis.

These findings are in line with those of Lee et al. [37] and James et al. [30], who reported that DOPA-functionalized copolymers showed no significant alteration in gelation behaviour when polymerised in the presence of atmospheric oxygen. Catechol-containing compounds like DOPA can oxidise to form reactive quinones, which promote crosslinking by converting hydroxyl groups to ortho-quinones. This process enhances cohesion and elasticity within the polymer network [30]. Although PDA had no significant effect on polymerisation, its chemical reactivity may contribute to improved long-term adhesive stability, though this was not directly assessed in the present study [30].

Contact angle measurements were performed using the sessile drop method to evaluate the wettability of unmodified and PDA-modified (2% and 4%) dentin bonding agents applied to etched dentin surfaces. Measurements were taken at 2 and 5 s. No significant differences were observed among the groups at 2 s. At 5 s, however, PDA incorporation significantly reduced the contact angle, indicating improved wettability of the dentin bonding agents in a time- and concentration-dependent manner. The 4% PDA-modified adhesive exhibited the lowest contact angle values, reflecting the highest surface hydrophilicity.

This progressive decrease highlights the contribution of PDA’s catecholamine groups (N-H and O-H), which increase surface polarity, reduce interfacial tension, and enhance interaction with the etched dentin substrate. The concentration-dependent effect observed at 4% PDA suggests that higher densities of hydrophilic groups provide greater spreading ability. Improved wettability at the adhesive–dentin interface is particularly advantageous, as it facilitates resin infiltration into the exposed collagen network and supports the formation of a more uniform hybrid layer. However, the influence of the improved wettability on bond strength was not evaluated in the present study. These findings are consistent with previous reports demonstrating that PDA increases the surface energy of biomaterials and enhances their interfacial performance in dental and biomedical applications [38, 39]. Although a dedicated contact angle goniometer provides higher temporal resolution and automated angle determination, the standardised video-based ImageJ method employed in this study enabled consistent comparison among the experimental groups under identical acquisition conditions.

The Vickers microhardness analysis provided quantitative insight into the mechanical response of the polydopamine (PDA)-modified adhesives following demineralisation. Following acid etching, all groups experienced a marked decrease in dentin hardness due to mineral loss and collagen matrix exposure. However, the extent of mechanical recovery after 7 days of storage in artificial saliva varied between the groups. In the control group, microhardness decreased significantly after etching and remained lower than baseline after 7 days, indicating limited recovery. The 2% PDA group also showed a significant reduction after etching, with 7-day values not differing significantly from either the baseline or the etched condition, consistent with partial recovery. The 4% PDA group followed a similar pattern; hardness decreased significantly after etching but approached baseline values after 7 days, suggesting improved mechanical recovery compared with the control. However, microhardness reflects surface resistance to indentation and does not directly confirm mineral deposition or its location within the dentin structure.

It should also be recognised that the measured hardness reflects the mechanical response of the adhesive–dentin complex rather than the dentin substrate alone. The polymerised adhesive layer and its thickness may contribute to the measured Vickers hardness values, although all specimens were prepared using a standardized adhesive application protocol to minimise variability. Therefore, the observed increase in hardness should be interpreted as an overall improvement in the surface mechanical properties of the adhesive–dentin interface rather than direct evidence of dentin remineralisation.

These findings suggest that PDA modification, particularly at 4%, may contribute to functional reinforcement of demineralised dentin rather than complete mechanical recovery. The relationship between mineral content and hardness is complex, and improvements in mechanical properties cannot be directly attributed to specific patterns of mineral deposition based on microhardness measurements alone. Previous studies have suggested that the distribution and integration of minerals within the collagen matrix play an important role in mechanical behaviour [40, 41]; however, such mechanisms were not directly assessed in the present study.

SEM imaging provided qualitative insight into surface morphology and mineral-like deposition across the experimental groups. The SEM observations were complemented by EDX analysis, which confirmed the presence of calcium and phosphorus within the surface deposits in all groups, supporting their calcium phosphate-containing nature. Representative EDX measurements demonstrated higher calcium and phosphorus contents in the PDA-modified groups, with the highest values observed in the 4% PDA group, consistent with the greater extent of mineral-like surface deposition observed by SEM. Nevertheless, SEM provides only surface morphological information, while EDX identifies elemental composition but does not determine the crystallographic phase or depth of mineral deposition. Therefore, the findings support the formation of calcium phosphate-containing surface deposits but should not be interpreted as definitive evidence of hydroxyapatite formation or true dentin remineralisation.

In the control group, mineral deposits were sparsely distributed, and the dentin surface exhibited features consistent with a demineralised, collapsed collagen structure with limited surface coverage. The observed deposits likely represent superficial mineral precipitation rather than structurally integrated minerals within the collagen matrix. In the 2% PDA group, an increase in mineral-like surface deposition was evident, with greater coverage and partial occlusion of dentinal tubules.

However, the deposits appeared heterogeneous and disorganised. The persistence of visible fibrillar structure suggests limited structural integration of the deposited mineral. The 4% PDA group exhibited more extensive and continuous surface deposits, with crystal-like morphologies observed at higher magnification, including needle- and plate-like structures. These observations, together with the higher calcium and phosphorus contents detected by EDX, suggest enhanced calcium phosphate nucleation on the dentin surface. However, neither SEM nor EDX can distinguish between inter- and intra-fibrillar mineralisation or confirm the crystallographic nature of the deposits.

The greater extent of mineral-like deposition observed in PDA-modified groups is consistent with the increase in microhardness and supports the hypothesis that PDA may facilitate mineral nucleation [42]. This effect may be related to the presence of catechol and amine functional groups, which have been reported to bind calcium and phosphate ions and promote calcium phosphate formation [43, 44]. However, these interactions were not directly measured in the present study and should therefore be considered as a proposed mechanism.

Self-etch adhesives interact with dentin based on their pH. Strong types (pH < 1) fully dissolve the smear layer, forming thick hybrid layers, while mild (pH > 2) and ultra-mild (pH ~2.7) adhesives cause shallow demineralisation, preserving more hydroxyapatite and creating thinner or nano-scale interaction zones [45]. pH measurements confirmed that PDA incorporation resulted in only small changes in the acidity of the adhesive system. Differences among groups were minimal and not considered practically significant, indicating that PDA modification preserves the etching functionality required for effective smear layer removal. The pH measurements represent the uncured adhesive formulations under static conditions. Potential changes in pH during polymerisation, after curing, or during ageing were beyond the scope of the present study and warrant further investigation.

The findings of this study should be interpreted within several limitations. All experiments were conducted in vitro using bovine dentin, which differs from human dentin in tubule density, tubule diameter, and mineral content. Although bovine dentin is a well-established and reproducible model for adhesive research [46], these differences may influence adhesive behaviour and mineral deposition. Therefore, caution should be exercised when extrapolating the present findings to clinical conditions. Mineral-related changes were assessed using microhardness and SEM, which provide indirect evidence of mineral deposition. Microhardness reflects mechanical recovery, whereas SEM assesses surface morphology. SEM analysis was qualitative and not performed under blinded conditions.

Moreover, the study did not evaluate key functional outcomes such as bond strength, nanoleakage, or long-term durability. The present study was designed to provide a comprehensive physicochemical characterization of PDA-modified dentin adhesives, including degree of conversion, wettability, pH, microhardness recovery, and mineral deposition potential. These properties provide important mechanistic insight into the behaviour of the modified adhesive. Evaluation of dentin bond strength is being conducted in a separate, complementary study to determine whether these physicochemical improvements translate into enhanced adhesive performance under clinically relevant conditions.

## Conclusions

This study focused on improving interfacial conditions rather than restoring the full mechanical function of dentin. Within the limitations of this in vitro study, incorporation of polydopamine (PDA) into a commercial dentin adhesive did not adversely affect polymerisation or acidity but influenced selected physicochemical properties. Specifically, incorporation of 4% PDA significantly enhanced dentin surface hydrophilicity, promoted calcium phosphate-containing surface deposition, and improved microhardness recovery following ageing in artificial saliva, while maintaining a clinically acceptable degree of conversion. These findings suggest the potential of PDA as a biomimetic functional modifier for commercial dentin adhesives by improving interfacial characteristics without compromising polymerisation efficiency. However, the results do not confirm true remineralisation or structural integration of mineral within dentin. Further studies, including bond strength testing, compositional analysis, and long-term in vivo evaluation, are required to validate these preliminary observations.

## Data Availability

The datasets used and/or analysed during the current study are available from the corresponding author upon reasonable request.
